# 208. Clinical Characteristics and Risk Factors for Septic Shock in Patients with Pyometra: A Retrospective Multicenter Cohort Study

**DOI:** 10.1093/ofid/ofad500.281

**Published:** 2023-11-27

**Authors:** Sukbin Jang, Minji Jeon, Seok Jun Mun, Si-Ho Kim

**Affiliations:** Dankook University School of Medicine, Cheonan, Ch'ungch'ong-namdo, Republic of Korea; Kosin University Gospel Hospital, Busan, Pusan-jikhalsi, Republic of Korea; Inje University Busan Paik Hospital, Inje University College of Medicine, Busan, Pusan-jikhalsi, Republic of Korea; Division of Infectious Diseases, Samsung Changwon Hospital, Sungkyunkwan University, Changwon, Korea., Changwon, Kyongsang-namdo, Republic of Korea

## Abstract

**Background:**

Pyometra is a disease of pus collection in the uterine cavity. The clinical characteristics and etiology of pyometra have not been well-described. In this study, we investigated the clinical characteristics, epidemiology, outcomes, and risk factors of septic shock in patients with pyometra.

**Methods:**

The study population was selected from adult patients (≥ 18 years old) diagnosed with the International Classification of Diseases 10th code for inflammatory disease of the uterus (N71) in four academic hospitals between 2010 and 2021. Only patients with definitive pus collection in the uterus confirmed by imaging tests were included. Intra-or post-partum infection and surgical site infection were excluded. The primary endpoint was all-cause 28-day mortality, and the secondary endpoint was a 1-year recurrence. Clinical characteristics and outcomes were compared among patients with or without septic shock.

**Results:**

Among 406 patients diagnosed with the N71 code, 193 patients were classified as pyometra. 28-day all-cause mortality was 5.0%, and the 1-year recurrence rate was 6.1%. The median patient age was 74.5 years. The most common symptom was abdominal pain (48.7%) and vaginal discharge (48.2%). The most common pathogens isolated from culture were *Escherichia coli* (39.9%), *Streptococcus* spp.(16.6%), and *Klebsiella pneumoniae*(16.0%) and isolated from polymerase chain reaction (PCR) were *Mycoplasma* spp., and *Ureaplasma* spp. Patients with septic shock were older and have a shorter interval between symptom onset (or discovery) and diagnosis. These patients had larger pyometra diameters, more frequent uterine perforation, more dementia, and were more frequently referred from long-term care facilities. The 28-survival rate was lower in patients with septic shock (72.8%) than without septic shock (98.7%, P < 0.001)

**Figure:**
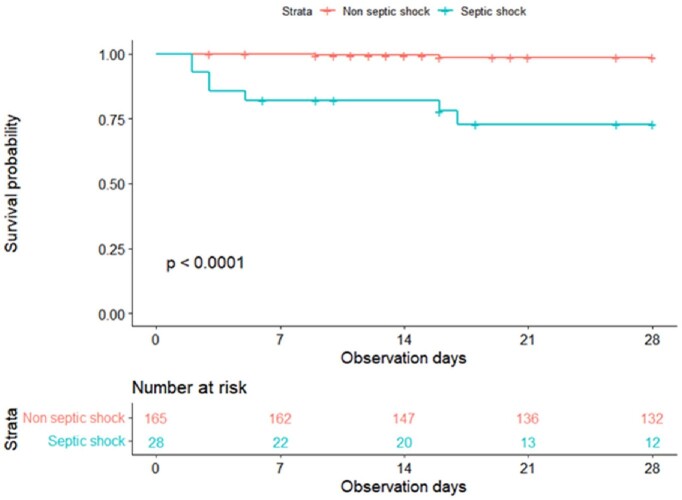

**Conclusion:**

Our study suggested pyometra is a unique gynecological infectious syndrome in post-menopausal women. The most common pathogen was similar to pathogens causing urinary tract infections. Risk factors with septic shock suggested that decreased cognitive functions could delay early diagnosis of pyometra, and lead to more septic shock and higher mortality.

**Disclosures:**

**All Authors**: No reported disclosures

